# Altered Resting-State EEG Microstate in Idiopathic Sudden Sensorineural Hearing Loss Patients With Tinnitus

**DOI:** 10.3389/fnins.2019.00443

**Published:** 2019-05-07

**Authors:** Yuexin Cai, Suijun Chen, Yanhong Chen, Jiahong Li, Chang-Dong Wang, Fei Zhao, Cai-Ping Dang, Jianheng Liang, Nannan He, Maojin Liang, Yiqing Zheng

**Affiliations:** ^1^Department of Otolaryngology, Sun Yat-sen Memorial Hospital, Sun Yat-sen University, Guangzhou, China; ^2^Institute of Hearing and Speech-Language Science, Sun Yat-sen University, Guangzhou, China; ^3^School of Data and Computer Science, Sun Yat-sen University, Guangzhou, China; ^4^Department of Speech Language Therapy and Hearing Science, Cardiff Metropolitan University, Cardiff, United Kingdom; ^5^Department of Hearing and Speech Science, Xinhua College, Sun Yat-sen University, Guangzhou, China; ^6^Affiliated Brain Hospital of Guangzhou Medical University, Guangzhou, China; ^7^Department of Psychology, Guangzhou Medical University, Guangzhou, China; ^8^College of Mathematics and Informatics, South China Agricultural University, Guangzhou, China

**Keywords:** EEG, microstate, tinnitus, ISSNHL, mechanism

## Abstract

In order to clarify the central reorganization in acute period of hearing loss, this study explored the aberrant dynamics of electroencephalogram (EEG) microstates and the correlations with the features of idiopathic sudden sensorineural hearing loss (ISSNHL) and tinnitus. We used high-density EEG with 128 channels to investigate alterations in microstate parameters between 25 ISSNHL patients with tinnitus and 27 healthy subjects. This study also explored the associations between microstate characteristics and tinnitus features. Microstates were clustered into four categories. There was a reduced presence of microstate A in amplitude, coverage, lifespan, frequency and an increased presence of microstate B in frequency in ISSNHL patients with tinnitus. According to the syntax analysis, a reduced transition from microstate C to microstate A and an increased transition from microstate C to microstate B were found in ISSNHL subjects. In addition, the significant negative correlations were found between Tinnitus Handicap Inventory (THI) scores and frequency of microstate A as well as between THI scores and the probability of transition from microstate D to microstate A. While THI was positively correlated with the transition probability from microstate D to microstate B. To sum up, the significant differences in the characteristics of resting-state EEG microstates were found between ISSNHL subjects with tinnitus and healthy controls. This study suggests that the alterations of central neural networks occur in acute stage of hearing loss and tinnitus. And EEG microstate may be considered as a useful tool to study the whole brain network in ISSNHL patients.

## Introduction

Sudden sensorineural hearing loss (SSNHL) is the sudden-onset and rapidly developed hearing impairment with reduction in hearing of at least 30 dB in no less than three consecutive audiometric frequencies within 72 h (Stachler et al., 2012). The incidence of SSNHL is approximately 5 to 20 individuals per 100,000/year (Klemm et al., 2009; Stachler et al., 2012). Some studies suggest that the hearing impairment of SSNHL is usually unilateral, and less than 5% of SSNHL patients is bilateral (Oh et al., 2007; Schreiber et al., 2010). SSNHL, whose etiology and pathogenesis are mostly unclear, is usually considered as idiopathic (Chau et al., 2010). No more than one third of cases are caused by viral infection, otologic disease, trauma, cardiovascular or hematologic risk factors, neoplastic disease, and autoimmune disease (Chau et al., 2010; Lin et al., 2012). At present, the main treatment for idiopathic sudden sensorineural hearing loss (ISSNHL) is steroid therapy (Jung et al., 2016). Unfortunately, many ISSNHL patients have little improvement of their hearing function even after high-quality suitable therapy, which may lead to the poor quality of their life (Kuhn et al., 2011). The discontented state of ISSNHL treatment may be due to the unclear mechanism of ISSNHL.

Idiopathic sudden sensorineural hearing loss is often accompanied by tinnitus, vertigo, ear fullness, and so on (Koç and Sanisoğlu, 2003). Among them, tinnitus is a perceived sound without any external sound source (Kang et al., 2017). Tinnitus is a common and annoying problem in ISSNHL patients. And the incidence of tinnitus in patients with ISSNHL is 80 to 95% (Rah et al., 2015; Nogueira-Neto et al., 2016). Tinnitus not only has a high incidence in ISSNHL patients, but also has a negative impact on the life of the sufferers. For example, ISSNHL patients with tinnitus have more depressive symptoms, mental distress, and disruptive personal relationship (Chen et al., 2013). As a result, physicians should attach great importance to the treatment of ISSNHL patients with tinnitus.

To cure ISSNHL subjects with tinnitus effectively, it is necessary to explore the intrinsic mechanism of ISSNHL completely. Previous researches have showed that ISSNHL is correlated with the deviant activity of some central neural networks (Seeley et al., 2009; Musiek et al., 2013; Wang et al., 2014; Zhou and Seeley, 2014; Fan et al., 2015; Zhang et al., 2015; Golden et al., 2016; Xu et al., 2016; Micarelli et al., 2017). The development of ISSNHL is associated with several function networks, including auditory network, default mode network (DMN) and limbic network (Xu et al., 2016; Micarelli et al., 2017). A prospective case research by Fan et al. (2015) suggested that subjects with unilateral SSNHL had significant decrease in gray matter of contralateral auditory cortex. Similarly, Micarelli et al. (2017) found that ISSNHL patients had the hypometabolism in the contralateral auditory cortex by Positron emission tomography (PET). The hypothesized mechanism behind ISSNHL may be related to the unbalanced activities between excitatory and inhibitory neurons, which led to the errors about the intensity perception at cortical level (Musiek et al., 2013; Fan et al., 2015; Micarelli et al., 2017). Moreover, Wang et al. (2014) found that the deficiency of unilateral sensory input changed the activity in sensory regions and reorganized the cognitive control network. A study by Zhang et al. (2015) suggested that long-term unilateral ISSNHL patients had an abnormal change in default node network, which might influence their cognitive abilities. In addition, the insula is an important brain region to link DMN with the networks which assess sensory information and program behavioral responses (Seeley et al., 2009; Zhou and Seeley, 2014). The decreased activity of insula is related to the inhibition of activity between DMN and auditory network (Golden et al., 2016). The increase of connection between auditory network and limbic system may be associated with the generation of tinnitus in ISSNHL patients (Xu et al., 2016). Although the deviant activity of several brain functional networks had been found in ISSNHL, more studies were needed to resolve the further central mechanism behind ISSNHL. This study aimed at exploring alterations in central nervous system of ISSNHL patients with tinnitus in a novel way.

Electroencephalography (EEG) is a high temporal resolution technique for exploring electrical activity of central brain areas. It records the electrical potentials within the brain through the electrodes on the scalp (Ingber and Nunez, 2011). So far, some ways can be applied to extract useful information from multichannel EEG data. Among them, microstate analysis explores multichannel EEG recordings across the scalp and defines a set of stable microstates (Khanna et al., 2015). Therefore, it not only partly compensates for the problem of low spatial resolution in EEG, but also analyzes the aberrant dynamics of the whole brain networks. As a result, we can acquire helpful information on brain networks by microstate analysis to further explore resting-state central neural activity. In general, microstates are clustered into four categories, including microstates A, B, C, and D. (1) Microstate A is related to activation in the bilateral superior and middle temporal gyrus regions that are correlated with phonological processing. (2) Microstate B is associated with bilateral visual cortex areas. (3) Microstate C is related to a salience network, which involved the detection and orientation of both internal and external stimuli. (4) Microstate D is associated with activation in the right-lateralized dorsal and ventral regions of the frontal and parietal regions that are correlated with the central executive network (Britz et al., 2010).

At present, several studies have suggested that some central neuropsychiatric diseases have significant changes in EEG microstates, including schizophrenia, dementia, depression, and panic disorder. According to the researches about these neuropsychiatric diseases, EEG microstate analysis may be an effective way to detect the objective indicators, recognize the disease’s severity, design the treatment plan and evaluate the treatment effect (Khanna et al., 2015). Additionally, EEG microstate has been used to study the neurophysiological mechanism of auditory hallucination and tinnitus. For instance, Kindler et al. (2010) found a significantly reduced presence of microstate D in hallucinating patients. Besides, Cai et al. (2018) suggested that microstate A was significantly longer and microstate D was significantly shorter in tinnitus subjects.

So far, knowledge of microstate abnormalities in ISSNHL is limited. To investigate the neurophysiological mechanism of ISSNHL, this study explores the changes in EEG microstates between ISSNHL subjects with tinnitus and healthy subjects. Additionally, it can confirm whether EEG microstate is a useful tool to investigate ISSNHL. With the alterations of nervous activities after ISSNHL and tinnitus, the hypothesis is that the significant alterations of microstates would be found in ISSNHL patients with tinnitus at rest. The significant result can be considered as a vital electrophysiological indicator of ISSNH.

## Materials and Methods

### Participants

The ISSNHL subjects with tinnitus were recruited from the Ear, Nose and Throat Clinic, Sun Yat-sen Memorial Hospital, Sun Yat-sen University, Guangzhou, China. Detailed selection criteria for inclusion and exclusion in the study were:

(1) Patients with at least 30 dB idiopathic sensorineural hearing impairment in no less than three consecutive audiometric frequencies within 72 h were recruited. In addition, ISSNHL was diagnosed by pure tone audiometry (PTA) within 72 h of conscious hearing loss or more than 72 h of conscious hearing impairment without appreciable progression of hearing damage.
(2) Patients had sought clinical help because of their hearing loss and tinnitus.
(3) Patients with acoustic neurinoma, autoimmune hearing loss, Meniere’s disease, depression, or insomnia were excluded.
(4) All patients with head trauma, central nervous system disorders, middle ear surgery or pulsatile tinnitus were also excluded.

There were 25 ISSNHL patients with tinnitus (9 males and 16 females; age Mean = 46.16 years, *SD* = 13.15 years) and 27 healthy controls (10 males and 17 females; age Mean = 41.48 years, *SD* = 13.53 years). A written consent form was signed by all participants after they had been properly informed about the background and purpose of the research. Our research was approved by the Institution Review Board of the Sun Yat-sen Memorial Hospital at Sun Yat-sen University of China.

### Routine Audiological Examinations

Routine audiological examinations included otoscopy and PTA. In the test of PTA, air conduction thresholds were measured at 125, 250, 500, 1000, 2000, 4000, and 8000 Hz for both ears, and bone conduction thresholds were measured between 250 and 4000 Hz in a sound-proof room. PTA was used to measure the hearing thresholds of all participants. In addition, the mean hearing threshold is defined as the average of hearing threshold at the frequencies of 0.5, 1, 2, and 4 kHz (British society of audiology recommendation, 1988; Dispenza et al., 2011; Bing et al., 2018).

### Tinnitus Specific Assessments

Patients were asked to describe the laterality of tinnitus. Then, tinnitus pitch matching test was performed (Teismann et al., 2011). In the process of tinnitus pitch matching measures, the tinnitus pitch was roughly matched through the use of nine hearing frequencies, including 0.125, 0.25, 0.5, 1, 2, 3, 4, 6, and 8 kHz. Participants contrasted the perceptive tinnitus pitch and the matching pitch, and then the matching pitch was adjusted to go higher or lower according to the feedback from patients. Ultimately, an exact or closely approximate matching pitch was gained by adjusting in a half-octave step. Narrow band noise was used if no matching pure tone was obtained from subjects.

Tinnitus Handicap Inventory (THI) was used to evaluate tinnitus severity. THI assessed the perceived level of handicap in tinnitus patients, according to a 0–100 increasing handicap scale (Newman et al., 1996). Participants were required to fill in the THI prior to the experiment.

### EEG Recordings

Electroencephalogram signals were recorded by a high-density EEG with 128 channels and were saved with Electrical Geodesics, Inc. (EGI, Eugene), and a NetAmps 200 amplifier. After understanding the purpose and requirements of the test, subjects were instructed to sit in a comfortable chair and remain calm. Then, participants were required to open the eyes and fixate a cross mark in front of them. A resting EEG for about 7 min was recorded. The sampling rate was set to 1000 Hz. The impedances of all electrodes were kept less than 50 kΩ and the CZ electrode was used as reference.

### Preprocessing of EEG Data

The raw EEG data was preprocessed by EEGLAB 13.0.0 in the toolbox of Matlab. Firstly, the data was re-referenced against the mean reference by all electrodes. The sampling rate of the data was reduced to 500 Hz/s. A notch filter was implemented at 50 Hz and the signals were band-pass-filtered from 0.5 to 80 Hz. Gross artifacts were manually removed by visual inspection. Some artifacts originated from one or a few distinct sources or a limited volume of space were removed. In addition, some artifacts characterized by a particular temporal pattern such as exponential decay were also removed. For example, in the pretreatment of resting EEG (eye-opening state), the largest artifact comes from eye electricity. Our system has a special electrode (E127 and E126) for recording eye electricity, but the effect of eye electricity is not limited to these two electrodes, it has a great influence on the electrode of frontal region. Therefore, we use ICA algorithm to correct it. The advantages of the ICA algorithm can be viewed in the literature (Onton et al., 2006), the artifact can be separated as independent components, and the other features of the original EEG signal can be effectively retained. Other artifacts, such as eye movement, muscle artifacts and heart beats, could be also removed by independent component analysis (ICA) correction. Then, the EEG signals were reconstructed and segmented into 2 s epochs. According to the study by von Wegner et al. (2018), the invariance of the quantities implies that the tested properties are algorithm-independent, and the microstate sequences are derived from inherent features of resting state EEG. Based on the previous research (Milz et al., 2016), more than 20 artifact-free epochs were used to microstate analysis randomly.

### EEG Microstate Analysis

Microstate analysis was done following the procedure described in the study by Cai et al. (2018). First of all, the global field power (GFP) was calculated by using the selected EEG epoch. Then, the clustering analysis was applied to the local maxima of the GFP. The GFP reflects the variation degree of potential across electrodes at a time point. The calculation formula is:

$$\text{GFP}(\text{t})=\sqrt{\frac{1}{\text{K}}\sum_{\text{i}=1}^{\text{K}}{\left({\text{V}}_{\text{i}}(\text{t})-{\text{V}}_{\text{mean}}(\text{t})\right)}^{2}}$$

Where, K is the number of electrodes in the EEG data. V_*i*_(t) is the potential of the *i*-th electrode at a certain time point. V_mean_(t) is the average value of the instantaneous potential across electrodes and the formula is V_mean_(t) = $\frac{1}{\text{K}}\sum_{\text{i}=1}^{\text{K}}{\text{V}}_{\text{i}}(\text{t})$. The EEG topographic maps at the local maxima of the GFP curve were taken as the local representative maps (Konig and Mellgarcia, 2009). Then, the clustering analysis was carried out to look for the stable microstates in EEG signals. All representative maps were divided into several types by the clustering analysis. It is showed that microstates can be clustered into four categories, namely microstate classes A, B, C, and D (Newman et al., 1996; Konig and Mellgarcia, 2009; Kindler et al., 2010; Teismann et al., 2011; Khanna et al., 2015; Cai et al., 2018). After obtaining the microstates of all participants, we compute the parameters of microstates, including topographical shape, amplitude, coverage, average lifespan, frequency of occurrence and transition probabilities of microstates (Cai et al., 2018). The amplitude of a microstate is the mean GFP that microstate is dominant. The coverage of a microstate is the percentage of recording time when the microstate is dominant. The average lifespan of a microstate is the average length of time when a microstate appears and remains stable. The frequency of occurrence of a microstate is the average number of times per second that a microstate occurs during a period of recording. The transition probability of a microstate means that the microstate is non-random and has the potential significance of sequence transfer.

### Statistical Analysis

SPSS 19.0 software was used for all statistical analyses. *t*-Test was performed to explore the significant differences of the microstate parameters between two groups. We applied Pearson correlation analysis to evaluate the correlation between microstate features and the characteristics of ISSNHL and tinnitus. A *p* level of less than 0.05 (two-sided) was considered to be statistically significant.

## Results

Table 1 shows the demographic information of ISSNHL subjects with tinnitus. No significant differences were found between the ISSNHL and control groups in terms of age (*t* = −1.263, df = 50, *p* = 0.212) and gender (x^2^ = 0.006, df = 1, *p* = 0.938). Table 1 also shows their hearing thresholds and tinnitus features, such as tinnitus laterality, pitch and duration.

**Table 1:** The landscape layout of ISSNHL participants with tinnitus.

Patient	Gender	Age (years)	Tinnitus duration (days)	Tinnitus laterality	Tinnitus pitch (Hz)	Hearing thresholds (dB HL) (L/R)	THI
CJH	Female	62	20	Left	125	95/19	34
CYF	Female	43	20	Left	4000	79/10	50
DL	Male	30	14	Left	8000	90/6	36
FLX	Female	61	7	Right	1000	31/81	58
HJW	Female	25	14	Right	1500	15/60	72
HDR	Female	63	7	Right	1000	53/112	78
HST	Male	42	7	Left	4000	80/25	26
LMJ	Male	54	14	Right	8000	25/110	62
LGZ	Female	58	10	Right	250	15/75	56
LJH	Male	51	20	Right	6000	47/56	26
LRH	Female	54	7	Right	6000	32/67	82
LWY	Female	49	7	Right	250	15/83	12
LSK	Male	46	25	Bilateral	2000/2000	58/63	26
LC	Male	47	2	Right	6000	19/81	12
LQ	Female	36	20	Left	125	110/9	58
MY	Male	41	7	Right	4000	38/68	4
PGJ	Male	51	20	Bilateral	4000/750	52/66	68
SWL	Male	42	10	Left	250	110/20	60
WJD	Female	57	20	Left	8000	110/22	52
WWM	Female	48	10	Right	1000	15/89	50
XWY	Female	17	14	Right	4000	8/81	16
ZMY	Female	18	10	Right	2000	20/83	48
ZYT	Female	41	8	Right	500	13/98	28
ZXH	Female	53	7	Left	6000	70/20	40
ZJH	Female	65	2	Right	4000	23/63	30

**FIGURE 1:**
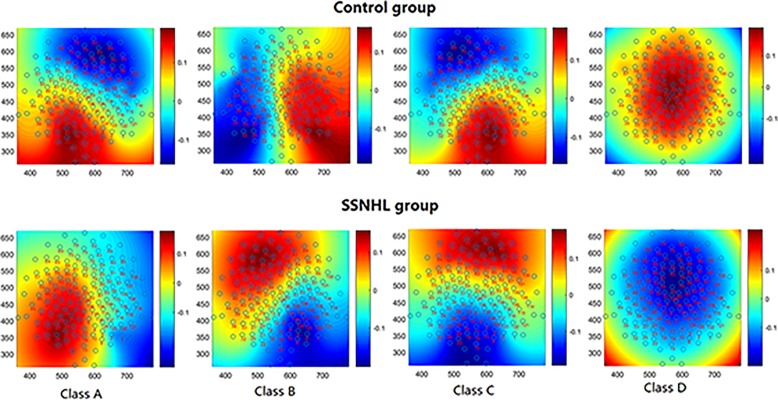
The spatial configuration of the four microstates classes in two group. Microstate class orientations were: **(A)** right-frontal left-posterior; **(B)** left-frontal right-posterior; **(C)** anterior–posterior; **(D)** fronto-central extreme.

Figure 1 shows the mean microstate topographies acquired from two groups. Similar to previous studies (Lehmann et al., 2005; Britz et al., 2010), four microstate maps were found and clustered into microstates A, B, C, and D.

**Table 2:** Comparison of the microstate analysis results in ISSNHL patients with tinnitus and control subjects.

	Class A		Class B		Class C		Class D	

	Control (Mean ± SD)	Tinnitus (Mean ± SD)	Control (Mean ± SD)	Tinnitus (Mean ± SD)	Control (Mean ± SD)	Tinnitus (Mean ± SD)	Control (Mean ± SD)	Tinnitus (Mean ± SD)
Coverage (%)	0.291 ± 0.227	0.149 ± 0.114^∗^	0.210 ± 0.241	0.296 ± 0.201	0.322 ± 0.235	0.371 ± 0.196	0.166 ± 0.212	0.179 ± 0.199
Lifespan (ms)	251.971 ± 142.799	170.686 ± 58.921^∗^	217.772 ± 175.857	247.793 ± 123.673	323.097 ± 315.186	307.430 ± 164.535	198.585 ± 128.154	190.289 ± 105.086
Amplitude (uV)	0.004 ± 0.081	−0.043 ± 0.087^∗^	−0.013 ± 0.310	0.015 ± 0.056	0.002 ± 0.062	−0.006 ± 0.031	−0.009 ± 0.169	0.059 ± 0.273
Frequency	1.022 ± 0.485	0.747 ± 0.448^∗^	0.715 ± 0.536	1.110 ± 0.360^∗^	1.045 ± 0.354	1.189 ± 0.272	0.615 ± 0.543	0.729 ± 0.513

*^∗^Indicates the t-test was significant (p < 0.05).*

Independent sample *t*-tests for microstate parameters between the ISSNHL (*N* = 25) and control (*N* = 27) groups are given in Table 2. The microstate parameters included the coverage, lifespan, amplitude, and frequency of the microstates. The significant differences in microstates A and B were found by the *t*-test. Compared with the control group, microstates A had significant decrease in coverage (*t* = 2.817, df = 50, *p* = 0.007), lifespan (*t* = 2.644, df = 50, *p* = 0.011), amplitude (*t* = 2.039, df = 50, *p* = 0.047), and frequency (*t* = 2.125, df = 50, *p* = 0.039). And microstate B had an increased presence (*t* = 3.096, df = 50, *p* = 0.03) in frequency for the ISSNHL group compared with the control group. There was no significant difference in microstates C or D (*p* > 0.05).

**FIGURE 2:**
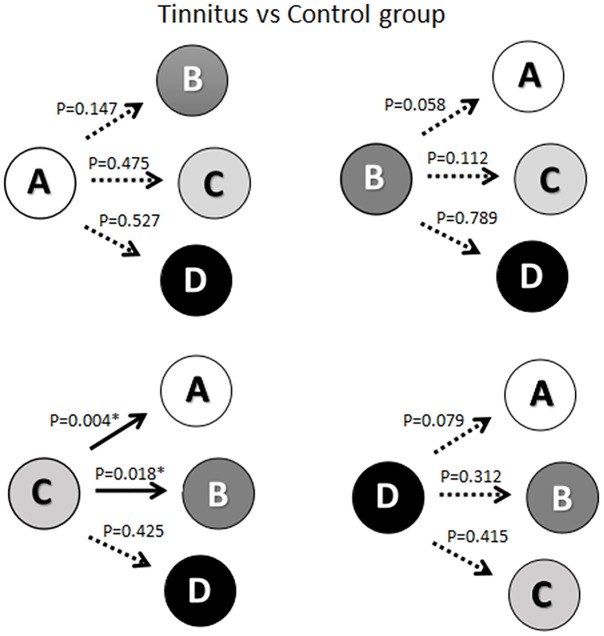
Schematic view of the syntax pattern. ^∗^ Indicates significant difference (*p* < 0.05). Solid arrow indicate significant correlation.

As shown in Figure 2, there was a significant reduction in the probability of transition from microstate C to microstate A (*t* = 2.978, df = 50, *p* = 0.004) in the ISSNHL patients compared with healthy subjects. Additionally, the probability of transition from microstate C to microstate B (*t* = −2.440, df = 50, *p* = 0.018) was increased significantly. No other significant difference in the probability of transition among microstates was revealed.

**FIGURE 3:**
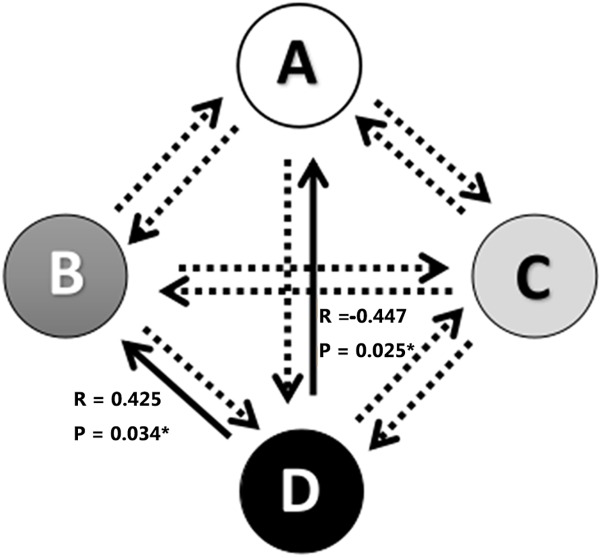
Correlations between transitions of microstates and tinnitus subjective symptoms. ^∗^ Indicates significant difference (*p* < 0.05). Solid arrow indicate significant correlation.

The associations between alterations in microstate parameters and tinnitus characteristics were shown in Figure 3 and Table 3. Significant negative correlations were revealed between THI scores and frequency of microstate A (*r* = −0.417, *p* = 0.038) as well as between THI scores and the probability of transition from microstate D to microstate A (*r* = −0.447, *p* = 0.025). While THI was significant positively associated with the transition probability from microstate D to microstate B (*r* = 0.425, *p* = 0.034). No significant correlation was revealed in the alterations of microstate characteristics with hearing threshold in ISSNHL subjects (*p* > 0.05).

## Discussion

As far as we know, it is the first research to explore the aberrant dynamics of EEG microstate in ISSNHL patients with tinnitus. The purpose of this research was evaluating the early stages of cortical plasticity after ISSNHL and tinnitus by using resting-state EEG microstate. There were significant differences in the temporal characteristics of the EEG microstate between ISSNHL patients and controls.

The coverage, lifespan, amplitude, and frequency of microstate A were significantly reduced in our study. Britz et al. (2010) demonstrated that microstate A was related to activation in the bilateral superior and middle temporal gyrus regions that were correlated with phonological processing. These results were similar to the finding of Fan et al. (2015), who found the decrease of contralateral auditory gray matter in unilateral ISSNHL. Additionally, Micarelli et al. (2017) also showed the decrease of the metabolism in contralateral auditory cortex in ISSNHL subjects. These interesting results indicated that cortical functional alterations already existed in ISSNHL subjects during acute period and mainly occurred in the auditory network.

**Table 3:** Correlations between changes of microstates and THI in ISSNHL patients with tinnitus.

THI scores	Microstate A R-value	Microstate B R-value	Microstate C R-value	Microstate D R-value
Coverage (%)	−0.358	0.197	−0.228	0.230
Lifespan (ms)	−0.235	0.137	−0.192	0.147
Amplitude (uV)	0.000	−0.411^∗^	0.053	−0.117
Frequency	−0.417^∗^	0.223	−0.193	0.257

*THI, Tinnitus Handicap Inventory; ^∗^ Statistical significance.*

Increase in coverage of microstate B was also revealed in the SSNHL subjects with tinnitus. A study by Britz et al. (2010) suggested that microstate B was associated with bilateral visual cortex areas. The increased coverage of visual network could indicate a compensatory mechanism secondary to ISSNHL. This result was similar to the finding by Campbell and Sharma (2013), who showed an alteration in cortical resource allocation with reduced activation in superior temporal gyrus and increased frontal activation in response to auditory input in hearing impairment subjects. These findings suggested that cortical reorganization facilitated visual and auditory cross-modal recruitment for visual processing in acute period after ISSNHL.

In current study, no significant difference was found in microstate C or microstate D. Britz et al. (2010) suggested that microstate C was related to a salience network, which involved the detection and orientation of both internal and external stimuli. Microstate D was related to signals in the right-lateralized dorsal and ventral regions of the frontal and parietal cortex. These regions roughly corresponded to the central executive network (Britz et al., 2010). This result was similar to finding by Fan et al. (2015), who only found decreased auditory cortical changes in ISSNHL patients. The reason may be the short-term of duration which is not sufficient to trigger changes of cognitive functions. However, the study by Yang et al. (2014) reported not only the decreased temporal cortical activation, but also the reduced gray matter volume in non-auditory brain areas such as posterior cingulate gyrus, precuneus, and right parahippocampal gyrus. Xu et al. (2016) also showed that alterations of limbic, paralimic, and auditory networks existed in ISSNHL. The differences might be due to the heterogeneity in sample characteristics including age, gender, hearing ability, laterality of hearing loss, educational level, and research methodology (Fan et al., 2015).

According to the syntax analysis, a significant reduction in the probability of transition from microstate C to microstate A was found. The reduced transition from the salience network to the auditory network was likely due to the deficiency of auditory sensory input. In addition, the increase was significantly found in the transition probability from microstate C to microstate B. This result indicated the compensatory plasticity after hearing loss with increased activity from the salience network to the visual network. ISSNHL subjects began to rely more on the visual information to compensate for their hearing impairment (Rouger et al., 2012; Campbell and Sharma, 2014).

Another interesting finding was the negative correlations between THI scores and the frequency of microstate A as well as between THI scores and the transition probability from microstate D to microstate A. While a positive association was found between THI scores and the transition from microstate D to microstate B. These results indicated that the severity of tinnitus was related to the central plasticity after ISSNHL with alterations of auditory network and changes of cognitive function (Chen et al., 2017). The decreased transition from executive attention network to auditory network and increased to visual network indicated that generation of tinnitus in acute period may be also due to disruption of auditory and non-auditory network (Elgoyhen et al., 2015; Song et al., 2015). However, Cai et al., 2018) cannot found significant correlation between THI scores and any microstate parameters in chronical tinnitus. The reasons may be relied on the difference of the tinnitus duration and the alteration of central reorganizaiton between acute and chronic periods for tinnitus subjects. Further longitudinal study was needed to explore the central characteristics of ISSNHL and tinnitus at each stage to clarify the mechanism of the generation and maintenance of tinnitus.

All in all, the results of this study showed that there was a reduced activity in auditory network and an increased activity in visual network. In addition, the negative correlation between THI scores and frequency of microstate A was found. The findings of this study provided the theoretical basis for the treatment of ISSNHL patients with tinnitus. For example, repetitive transcranial magnetic stimulation (rTMS) is a non-invasive way that can modulate the excitability of the brain cortex (Formanek et al., 2018). And rTMS targeted on the abnormal central networks may be a strategy for the treatment of tinnitus. According to the findings of our study, we can improve the excitability of brain regions of auditory network by using rTMS, which may be beneficial for the improvement of symptoms of ISSNHL patients with tinnitus. Additionally, THI scores of acute tinnitus may reduce with the excitement of auditory network. More further studies were needed to explore the clinical effective treatment by using the findings of tinnitus associated researches.

Nonetheless, it is necessary to realize that ISSNHL is a heterogeneous disease with different hearing function, laterality and concomitant symptoms, such as tinnitus and vertigo (Fan et al., 2015). Therefore, it is hard to eliminate its heterogeneous factors completely even with the use of strict inclusion and exclusion criteria. Additionally, this study investigate aberrant dynamics of EEG microstates in ISSNHL patients with tinnitus and cannot separate the effects of acute tinnitus and ISSNHL on central nervous system respectively. Because of the perspectiveness and novelty of the study, our research has some limitations and its results need to dispose with caution. A further research with larger sample sizes and more subgroups comparisons is needed to clarify the central mechanism of ISSNHL and tinnitus in acute period.

## Conclusion

There were several significant differences in temporal characteristics and syntax of EEG microstates between ISSNHL patients with tinnitus and the controls at rest. This study suggests that the alterations of central neural networks occur in acute stage of hearing loss and tinnitus. EEG microstate may be considered as a useful way to explore the brain network in ISSNHL patients with tinnitus.

## Data Availability

The datasets generated for this study are available on request to the corresponding author.

## Ethics Statement

This study was carried out in accordance with the recommendations of the Institution Review Board of the Sun Yat-sen Memorial Hospital with written informed consent from all subjects. All subjects gave written informed consent in accordance with the Declaration of Helsinki. The protocol was approved by the Institution Review Board of the Sun Yat-sen Memorial Hospital.

## Author Contributions

YxC and SC conceived and designed the research. YhC, JoL, and C-DW performed the experiments. JeL, NH, C-PD, and ML responsible for the data analysis. FZ, YxC, and YZ drafted the manuscript. All authors had a lot of contributions at all stages of preparing the manuscript.

## Conflict of Interest Statement

The authors declare that the research was conducted in the absence of any commercial or financial relationships that could be construed as a potential conflict of interest.
